# Personalizing Seizure Detection for Individual Patients by Optimal Selection of EEG Signals

**DOI:** 10.3390/s25092715

**Published:** 2025-04-25

**Authors:** Rosanna Ferrara, Martino Giaquinto, Gennaro Percannella, Leonardo Rundo, Alessia Saggese

**Affiliations:** Department of Information Engineering, Electrical Engineering and Applied Mathematics (DIEM), University of Salerno, 84084 Fisciano, Italy; roferrara@unisa.it (R.F.); pergen@unisa.it (G.P.); lrundo@unisa.it (L.R.); asaggese@unisa.it (A.S.)

**Keywords:** channel selection, EEG analysis, lightweight CNN, personalized medicine, seizure detection, wearable systems

## Abstract

Electroencephalography is a widely used non-invasive method for monitoring brain electrical activity, critical for diagnosing and managing neurological disorders such as epilepsy. While clinical standards use 21 electrodes to capture comprehensive neural signals, a personalized approach can enhance performance by selecting patient-specific channels, reducing noise and redundancy. This study introduces an innovative, lightweight deep learning system optimized for real-time seizure detection in personalized wearable devices. The system uses an efficient Convolutional Neural Network that processes data from just two channels. These channels are automatically selected using a data-driven mechanism that identifies the most informative scalp regions based on each patient’s unique seizure patterns. The proposed approach ensures high reliability, even with small datasets, and improves interpretability for clinicians by overcoming the limitations of more complex methods. The tailored channel selection boosts detection accuracy and ensures robust performance across different seizure types while reducing the computational burden typical of multi-electrode systems. Validation on the publicly available CHB-MIT dataset achieved an average balanced accuracy of 0.83 and a false-positive rate of approximately 0.1/h. The system’s performance matches, and in some cases outperforms, state-of-the-art systems that use four fixed channels in temporal regions, demonstrating the potential of two-channel wearable solutions, specifically with a non-negligible 30% reduction in the false-positive rate. This interpretable, patient-specific method enables the development of personalized, efficient, and compact wearable devices for reliable seizure detection in everyday life.

## 1. Introduction

Wearable devices are revolutionizing healthcare by enabling continuous, real-time monitoring of physiological parameters. Modern technology now offers off-the-shelf solutions fully integrated into everyday items, such as smartwatches and sensorized clothing, capable of monitoring cardiovascular, respiratory, and motion-related bio-signals [1]. The key enabling technology in this field is Artificial Intelligence (AI), enabling efficient diagnosis support through real-time data analysis, even on portable low-power and low-consumption platforms [2], empowering both specialists and automated systems to deliver targeted, patient-specific therapies, aligned with the new vision of *personalized medicine* [3]. For example, glucose sensors connected to insulin pumps have become crucial to ensure a regular life even for diabetic patients [4].

Despite these advances, the application of wearable technology to electroencephalography (EEG) remains a significant challenge. EEG signals are indeed characterized by high diversity and variability generated by the electrical activity of neurons and acquired by means of electrodes embedded in a headset placed across the scalp [5]. EEG is widely used in fields such as emotional state monitoring [6], brain–computer interfaces [7], sleep studies [8], rehabilitation for neurological disorders [9], and epileptic patient monitoring [10]. Among these, monitoring epileptic patients during their daily activities is certainly among the most important applications in which a wearable EEG could play a crucial role. EEG-based seizure detection faces two main challenges: the complexity of EEG signals and the difficulty of designing a wearable, non-invasive, and practical device for everyday use [5]. To address these challenges, different research groups have focused their efforts on reducing the number of electrodes required, making the system less invasive and more feasible for daily use. Several solutions have been proposed, including wearable devices integrated into eyeglasses [11] or positioned behind the ear [12,13]. Following this trend, *four temporal channels* were recently used to define various algorithms tailored for microcontroller units, including a transformer-based model [14] and a Convolutional Neural Network (CNN) [15] with performance comparable to that obtained by a standard 10–20 system based on 21 channels [16]. However, seizures can be generalized or focal onset, with focal seizures often originating in specific regions of the brain, but highly variable and dependent on the specific patient [17]. This variability complicates optimal channel selection, especially for focal seizures, which account for approximately 60% of cases. Therefore, although these methods based on four fixed channels allow for generalizing the technical solution based on implementation needs, while also improving patient comfort, they may result in sub-optimal channel selection from an accuracy point of view, especially when focal seizures occur in regions not covered at all by the selected channels.

This is why several data-driven approaches have also been employed to select channel subsets that achieve detection performance comparable to *full-channel* systems. These approaches implement well-known feature selection methods, commonly categorized into (*i*) filtering, (*ii*) wrapper, and (*iii*) embedded methods [18]. *Filtering methods* rank channels using statistical or information theory criteria (e.g., correlation, variance, and entropy). They are computationally efficient, classifier-independent, and highly interpretable, making them well suited for clinical use since the selected features are easily understandable by medical experts [19,20]. However, they may not always capture complex inter-dependencies between channels. *Wrapper methods* identify channel subsets by training classification models—e.g., Random Forests (RFs) and K-nearest neighbors (KNNs)—in iterative search processes, typically achieving higher accuracy than filtering methods. However, they are computationally demanding and prone to overfitting, especially with small datasets, limiting their practicality [21]. *Embedded methods* combine feature selection with model training through regularization techniques like the multi-head attention mechanism [22] or LASSO [23], balancing performance and computational cost. Although more efficient than wrapper methods, they are restricted to models that support integrated selection, reducing their general applicability [24].

Due to the above mentioned limitations, no single objective, reliable, and automated method has emerged as the definitive solution for channel selection, making the expertise of clinicians crucial for refining channel selection in practical settings. Studies aiming at reducing the number of channels to fewer than three [19,20,21,22], in fact, often are based on expert-guided visual methods, where neurologists rely upon domain knowledge and optimize channel selection based on each patient’s unique seizure location [25].

**Figure 1:**
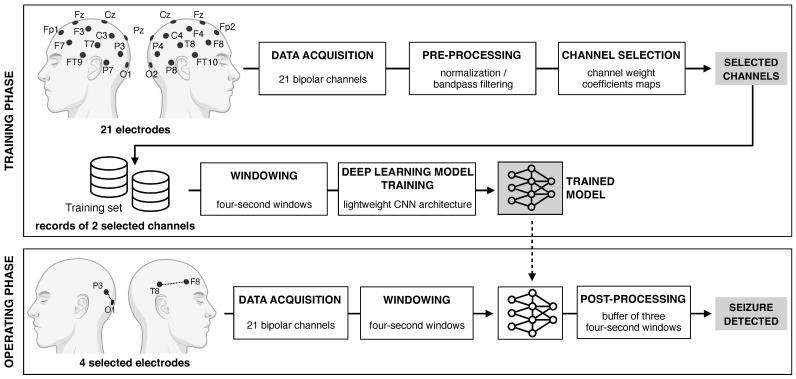
Overview of the proposed method. The overall workflow is divided into training and operating phases. During the training phase, EEG data are collected from 21 bipolar channels, preprocessed, and analyzed to identify the most relevant channels for the single patient using PCA. A lightweight CNN model is then trained on 4 s windowed segments of EEG data from these selected channels. The operating phase involves acquiring and segmenting data into 4-s windows from only two selected bipolar channels in the test set. The trained model is then used in inference to detect seizure onsets, also exploiting post-processing techniques.

In this framework, we present an efficient deep learning approach to design a next-generation wearable EEG system that combines personalization and advanced data processing in an ultra-lightweight architecture (Figure 1). Our solution features a highly optimized CNN capable of delivering state-of-the-art seizure detection in real time, even on low-power microcontroller units, ensuring both portability and efficiency. The system is tailored to each patient’s unique seizure characteristics, relying on an automatic channel selection that reduces the traditional multichannel EEG setup to only two highly informative channels, without compromising diagnostic accuracy. At the core of this method lies a classifier-independent and data-driven channel selection strategy, which assigns a weight coefficient to each channel. This approach preserves the interpretability and reliability typical of traditional filtering methods while going further by exploiting an eigen-decomposition that accounts for inter-channel relationships. The weight coefficients dynamically highlight the most informative channels during the different seizure phases, ensuring clinically meaningful insights and an intuitive selection process. The simplicity and robustness of the proposed approach make it particularly well suited for small datasets, such as pre-recorded EEG data from epileptic patients, where more complex models risk overfitting. By integrating deep learning innovations with an optimized data selection strategy, our method captures the most relevant information while minimizing computational overhead. This enables a highly efficient, personalized seizure detection system that can be seamlessly embedded in non-invasive wearable devices, facilitating real-time, patient-specific monitoring. We validate this approach on a widely used publicly available benchmark dataset [26], showing that personalized electrode reduction maintains, and in some cases improves, seizure detection accuracy. Our results indeed demonstrate performance comparable to or exceeding that of standard multichannel systems, paving the way for practical, non-invasive wearable EEG solutions.

To summarize, our main contribution concerns the development of personalized wearable devices for EEG-based seizure detection: the devised patient-specific, data-driven, and time-aware channel selection approach leverages the two most informative EEG channels for a downstream seizure detection model based on a lightweight CNN.

## 2. Methods

An overview of the overall workflow is shown in Figure 1. The process involves the following: raw data acquisition from an EEG dataset and pre-processing, patient-specific channel selection, windowing, training of a lightweight CNN classification model (at training time), classification of data, and post-processing (at operating time). The model classifies each window as either inter-ictal (class 0) or ictal (class 1). The classification of multiple consecutive windows enables the detection of epileptic ictal events.

### 2.1. Dataset

For experimental purposes, we used the publicly available Children’s Hospital Boston–Massachusetts Institute of Technology (CHB-MIT) scalp EEG dataset [26]. The dataset contains 686 records acquired with a sampling frequency of 256 Hz from 24 pediatric patients (5 males aged 3–22 years, 18 females aged 1.5–19 years, and 1 individual with unknown age and gender). In total, the dataset provides more than 950 h of records acquired from bipolar montage channel configurations, in accordance with the 10–20 international system [27]. The dataset includes, for each patient, records acquired during their normal state as well as records obtained during seizure events. The latter are annotated with the start and end of the ictal events, providing the ground truth for a classification problem with two classes: ictal, representing the period of the ictal event, and inter-ictal, representing the period between the end of one ictal phase and the onset of the next. Notably, the dataset contains data from patients with both generalized and focal seizures, even if the specific seizure type is not specified.

### 2.2. Pre-Processing

All the records were cleaned of the 60 Hz component using a 1 Hz wide notch filter (two-order Butterworth filter) and then normalized using the *z*-score method, i.e., by subtracting the mean and dividing by the standard deviation to obtain zero mean and unitary standard deviation. After normalization, the EEG signals were filtered to extract the following standard frequency ranges, δ[0.5,4]Hz, θ(4,8]Hz, α(8,13]Hz, β(13,35]Hz, and γ(35,80]Hz, using a two-order Butterworth bandpass filter.

### 2.3. Channel Selection Procedure

The first goal of our analysis, which can also be considered an important contribution of this work, is to identify the scalp regions from which to retrieve the most informative content during different phases of epileptic seizures. In turns, this allows for determining the optimal placement of EEG electrodes for a compact wearable device. This task is challenging due to the transient nature of EEG patterns, their high susceptibility to noise and artifacts, and the limited size of most EEG datasets, which increases the risk of overfitting when applying complex algorithms. To address these difficulties, we propose a method based on temporal Principal Component Analysis (PCA) to rank EEG channels based on their contribution to seizure-related activity. Each channel corresponds to a specific scalp region according to the 10–20 standard system. By decomposing the data into orthogonal, uncorrelated new variables, this approach highlights the most informative channels while suppressing noise and redundancy, making it particularly suitable for small EEG datasets, typically acquired in clinical practice. The procedure adopted is defined in three main steps, as described in the following.

#### 2.3.1. Channel Weight Coefficient Definition

The 21-channel EEG signal was segmented into 1-s time windows for each frequency band considered. Since the signal was sampled at 256 Hz, each time window was represented as a matrix X of size N×C, with N=256 being the number of samples per second and C=21 the number of bipolar EEG channels. This segmentation step allowed for a more localized analysis of neural activity over time, ensuring that transient seizure patterns could be captured. The generic matrix X was then decomposed, as follows, into principal components (PCs) that capture the main patterns of variation in the data:

(1) Y=XL

$L\in R^{C\times C}$ is the loading matrix, whose columns are the eigenvectors of the covariance matrix $S_{X}$ of the original data X [28]. Each *j*-th column of L represents the orientation of ${\mathrm{PC}}_{j}$ relative to the original EEG channels. Its elements, $l_{ij}$, known as *loadings*, quantify the contribution of the *i*-th EEG channel to the corresponding ${\mathrm{PC}}_{j}$.

The loading matrix was used here to rank the channels based on their contribution to the PCs. However, loadings alone do not indicate how much of the total variance each component explains. Instead, this information is provided by the covariance matrix of the transformed data Y, given by the following:

(2) $$S_{Y}=L^{⊤}S_{X}L$$

$S_{Y}$ is a diagonal matrix whose elements $s_{Yj}$ are the *eigenvalues*, corresponding to the variance explained by the *j*-th PC. PCs with larger $s_{Yj}$ values correspond to dominant neural activity patterns, whereas those with smaller values mainly capture noise or minor fluctuations. As a result, seizure-related activity, which is often localized to specific brain regions, are expected to be reflected by PCs that account for the greatest portion of the data variance. To integrate the explained variance into the channel ranking process, we weighted the absolute values of the loadings by the corresponding eigenvalues:

(3) $$l_{ij}^{\mathrm{weighted}}=\left|l_{ij}\right|\cdot s_{Yj},\;\mathrm{for}\;i,j=1,2,\ldots ,C.$$

This approach ensures that the most relevant seizure signatures are prioritized over less informative background activity, which is crucial for diagnostic purposes. Finally, to obtain a compact representation for each 1-s window, we summed the weighted loadings for each EEG channel to produce a single weighted mean coefficient for each channel, hereinafter defined as the channel weight coefficient (CWC):

(4) $${\mathrm{CWC}}_{i}=\sum_{j=1}^{C}l_{ij}^{\mathrm{weighted}},\;\mathrm{for}\;i=1,2,\ldots ,C.$$

This transformation reduces each 1-s multichannel segment to a compact vector of CWC values, with each value representing the weight of a specific channel in expressing the relevant information content. This provides us with a tool to select the most informative channels within different phases, rather than reducing the data dimensionality by exploiting the linear combination of original channels, as performed in previous work employing the PCA for EEG data [29,30]. It is important to remark that z-score normalization ensures that all channels are on a comparable scale, preventing variables with larger numerical ranges from disproportionately affecting the PCs. Moreover, as this procedure is explicitly based on the eigen-decomposition of the covariance matrix, it inherently captures the linear correlation structure among the analyzed variables (i.e., the original EEG channels). Therefore, this method allows for the selection of the most statistically informative and mutually uncorrelated EEG channels, giving the same importance to the informative content within the clinical frequency bands.

#### 2.3.2. Channel Weight Coefficient Evolution over Time

The procedure described in Section 2.3.1 was repeated for all 1 s segments within a specific record, resulting in the temporal evolutions of the CWCs. Tracking the evolution of CWCs over time allows us to monitor changes in neural activity across seizure phases, identifying scalp regions where activity is concentrated during ictal events. This provides valuable insight into seizure propagation patterns and also serves as an effective tool for the definition of a channel selection algorithm. Consequently, each phase could be effectively represented by one channel, i.e., the one with the highest CWC. Given that our task addresses a binary classification problem between ictal and inter-ictal, we specifically focus on the subset of channels that are most representative of these two phases. To further enhance the distinction between the classes, we limit the inter-ictal phase to a reduced subclass, as will be better described in Section 3.1. Therefore, such a temporal analysis enables the identification of the most informative channels at each phase of interest, aiming at improving the detection performance.

#### 2.3.3. Automated Selection Algorithm

To define a fully automated channel selection algorithm, it was useful to compress the information into a single coefficient per channel for each phase, merging information from the different bands. This yielded 21 values for the inter-ictal and 21 for the ictal phase. With this aim, the CWCs representative of each phase were first normalized into the [0,1] range by using a min–max normalization within each frequency band, thus preserving information from all frequency bands and ensuring equal contribution from all of them. These normalized frequency-dependent CWCs were then averaged across frequency bands (denoted by the set B), yielding average CWCs (represented as $\bar{\mathrm{CWC}}$) for each phase:

(5) $${\bar{\mathrm{CWC}}}_{i}=\frac{1}{\left|B\right|}\cdot \sum_{b\in B}{\mathrm{CWC}}_{i}^{\left(b\right)},\;\mathrm{with}\;i=1,2,\ldots ,C.$$

In our case, we consider the five standard frequency bands B={δ,θ,α,β,γ}. Finally, since each patient dataset contains multiple records, this procedure was repeated for each of them, and the results were averaged.

As a final step, we identified a pair of channels as the most informative in each of the two phases (ictal and inter-ictal), specifically those with the highest average weight. To maximize the difference in information content between the selected channels and minimize redundancy, we ensured that the channels were not dominant in both maps, allowing each to contribute with unique and non-overlapping information. This selection procedure can be fully automated using the following two-step algorithm: (*i*) digitize the average maps by normalizing the values into a [0,1] scale, assigning a value of 1 to values above a threshold (experimentally set to 0.9) and 0 to others, and (*ii*) perform an XOR) operation on the two digitized values sets.

It should be noted that the selection algorithm may identify multiple representative channels for each phase if several channels exceed the $\bar{\mathrm{CWC}}$ threshold. While this may provide insights into seizure characteristics, it also necessitates selecting an optimal channel pair for classification. To refine this selection, we introduce a stronger criterion based on inter-channel correlation. In particular, we define an average correlation coefficient ($\bar{\rho }$) in the range [0,1], based on the absolute Pearson correlation coefficient (PCC) [31]. The PCC values were computed over 1-s sliding windows across frequency bands and averaged to obtain $\bar{\rho }$. This coefficient helps discriminate between channel pairs when multiple options arise by selecting the least correlated pair and also serves as an interpretative tool in the experimental phase.

### 2.4. Deep Learning Model Definition and Training

#### 2.4.1. Neural Network Architecture

CNNs are widely used for seizure detection in EEG signals due to their ability to automatically learn hierarchical features from raw data. Recent advancements have addressed challenges related to the resource limitations of wearable devices, such as low-power microcontrollers, by optimizing models for energy efficiency. Qiu et al. introduced LightSeizureNet [32], which applies dilated 1D convolution to each of the 21 channels in a standard EEG system, utilizes global average pooling, and employs kernel-wise pruning to compress the model. This approach reduces computational complexity while maintaining high accuracy and provides fine-grained information, such as activated brain regions and frequency bands during seizures, supporting the physician in the clinical diagnosis.

Similarly, Zhao et al. proposed a Neural Architecture Search method to design a compact CNN architecture with approximately 11,000 parameters, achieving high sensitivity with energy consumption in the millijoule (mJ) range per inference [16]. The network consists of five convolutional blocks, each of them including a convolutional and a pooling layer to capture essential temporal and spatial features of EEG signals. Ingolfsson et al. refined this architecture by tailoring it to four input channels, further reducing the computational load and power consumption [15]. Their EpiDeNet architecture was then chosen in our work as a solid baseline due to its lightweight design and proven effectiveness, both in terms of classification performance and real-time metrics, such as inference time and power consumption. In its original four-channel configuration, in fact, the network achieved an inference time of 2.84 ms on a GAP9 platform (RISCY core at 240 MHz), with a power consumption of just 17.89 mW [15]. Although performance slightly decreases on less efficient platforms, it still maintains values suitable for daily wearable use. For instance, on an STM32 Cortex-M7 running at 216 MHz, the inference time increases to 31.99 ms, with the power consumption rising to 413.03 mW.

Building upon this approach, we propose further architectural optimization by introducing batch normalization (BN) layers after each convolutional block, aimed at facilitating and accelerating network convergence [33]. Each BN layer normalizes the corresponding feature maps using a number of parameters equal to the number of output channels produced by the preceding convolutional layer. Moreover, we adapted the network structure to effectively handle the reduced input dimensionality by tailoring the shape of the spatial convolutional filter. The architecture is summarized in Table 1. It includes five convolutional blocks designed to extract spectral characteristics through frequency filters and recognize spatial patterns contingent on specific frequency bands through spatial filters. Each block consists of a 2D convolutional layer, designed to keep the output with the same shape of input, via padding. This is followed by BN, a ReLU activation function, and a max pooling layer. The first pooling layers are designed to reduce the temporal dimension, with kernel sizes having a height of 1 and widths set to 8, 4, and 4, respectively. The final max pooling layer has a fixed width of 1 and a height that adapts to the input configuration, denoted as *M*. The final max pooling layer has a fixed width of 1 and a height that corresponds to the number of input channels to the network, denoted as *M*. Specifically, *M* is set to 2 in the proposed two-channel configuration with an input size of 2×1024 and to 4 in the four-channel configuration with an input size of 4×1024. The final part of the architecture includes a global average pooling layer and a fully connected dense layer with a softmax output function.

The adopted neural network was implemented using the PyTorch framework (version 2.1.2) with CUDA 12.1 support. All the training and testing procedures were performed on a workstation equipped with an NVIDIA Tesla V100 DGX GPU. The source code is available on GitHub: https://github.com/MiviaLab/INBIT/tree/main/Seizure-detection.

#### 2.4.2. Patient-Specific Training Strategy

With reference to the schematic represented in Figure 1, the proposed workflow is based on an initial selection of effective channels and the training of the CNN relying on the availability of EEG data recorded using a standard acquisition system over a period in which at least one seizure occurs (e.g., in a clinical setting). The standard acquisition systems should allow for obtaining signals from the whole scalp (for example, by using 21 channels of the standard 10–20 system) [5]. It is important to remark that this approach reflects the typical clinical workflow where seizure events are recorded during diagnostic EEG monitoring sessions (e.g., in hospital or clinical scenarios) [12]. In other words, for an epileptic patient, there is typically the availability of EEG records acquired during seizure events. The PCA-based method described before is used to identify the two most informative channels for that specific patient, across ‘ictal’ and ‘no-seizure’ phases. These two channels are then used to train the CNN model. Once this personalized model is trained, the user can transition to a wearable, real-time monitoring setup using only the two selected EEG channels. This makes the system highly efficient and suitable for long-term, at-home use with minimal hardware complexity.

Within the framework of personalized medicine, this study aims to identify the optimal wearable device configuration tailored to each individual patient. This involves not only determining the most effective electrode placement but also selecting a model specifically trained to the patient’s unique characteristics. To accomplish this, a leave-one-record-out (LORO) cross-validation approach was adopted [14]: for each patient, the model was trained on all the available records except one, which was reserved for testing, while the remaining records were split between training and validation sets. This procedure was repeated for each record, ensuring that the model was exposed to all available intra-patient patterns, including the various physiological phases that may characterize inter-ictal states [34]. Note that we run the LORO scheme across all the records available for each patient, including those not containing ictal annotation, thus assessing the system robustness against false alarms.

It is worth mentioning that due to the intrinsic rarity and short duration of ictal events compared to the inter-ictal phases, the dataset is characterized by an inherent class imbalance. The class imbalance ratio is very variable, ranging from 86:4310 to 47:47,234. Thus, to mitigate this problem, each batch is composed of 64 samples, 5% of which (when available) belong to the minority class (namely, the seizure detection). In this way, we ensured that the model was consistently exposed to examples of the minority class throughout the training process. This approach avoids the need for minority class over-sampling or synthetic data generation and is particularly effective when combined with patient-specific training, as in our framework.

The training phases run for at most 150 epochs and the best models were saved according to the loss registered on the validation set. In order to avoid overfitting on the training data, we stop the training if the performance on the validation set does not improve for ten consecutive epochs. We employed the cross-entropy loss function, with the Adam optimizer characterized by $\beta_{1}=0.9$, $\beta_{2}=0.999$, and a learning rate of $10^{-3}$.

Performance was evaluated for each test record, with the results presented as the mean and standard deviation calculated across all the available records for each patient, in accordance with the evaluation metrics outlined in the following section.

#### 2.4.3. Evaluation Metrics

The model performance achieved on each 1-h test record was evaluated at the *segment level*, through a post-processing phase by considering a *segment* defined as a buffer of three consecutive four-second sliding windows, with a two-second overlap, classified as ictal or inter-ictal [20]. In more detail, the evaluation metrics used to assess detection performance included sensitivity (SN), which is the number of true ictal segments of three consecutive four-second windows (TP) with respect to the total number of actual ones [35]:

(6) $$\mathrm{SN}=\frac{\mathrm{TP}}{\mathrm{TP}+\mathrm{FN}},$$

with FN being the number of false-negative segments. This metric is an important indicator of the model’s ability to accurately identify ictal occurrences. In addition, we also considered specificity (SP), which measures the proportion of true inter-ictal segments identified correctly, namely, the true negative (TN), among the total actual inter-ictal instances [35]:

(7) $$\mathrm{SP}=\frac{\mathrm{TN}}{\mathrm{TN}+\mathrm{FP}},$$

where FP denotes the number of false-positive segments.

Along with accuracy (Acc), defined as follows:

(8) $$\mathrm{Acc}=\frac{\mathrm{TP}+\mathrm{TN}}{\mathrm{TP}+\mathrm{TN}+\mathrm{FP}+\mathrm{FN}},$$

we considered the balanced accuracy (bAcc), which accounts for the imbalanced nature of the dataset by calculating the arithmetic mean of the sensitivity and specificity values:

(9) $$\mathrm{bAcc}=\frac{1}{2}\left(\mathrm{SN}+\mathrm{SP}\right).$$

Evaluating performance at the segment level with the described approach is more restrictive than the majority-voting method applied over three consecutive windows described in previous studies [15]. The use of this stricter post-processing criterion helps to mitigate the effect of FPs.

In addition, to further evaluate the system’s usability, we also derive additional performance metrics evaluated at the *event level*, where *event* defines the whole ictal phase detected by the model. Actually, an event includes consecutive segments classified as ictal. First, to gain a clearer understanding of the system’s ability to detect ictal events, we consider the effective number of correctly identified ictal events (with respect to the total number of ictal events).

Moreover, we also analyze the detection latency, defined as the time difference between the annotated ictal onset and the model’s detection instant, hereinafter referred to as *delay*, expressed in seconds. Finally, it is important to consider that a system that generates false alarms will lose user trust, so it is crucial to understand how FPs affect reliability. For this reason, we define the FP rate as the average number of FP events detected per hour (FP/h) [36].

## 3. Results

The experiments were carried out using a patient-specific approach to assess the value of a personalized wearable device. Performance was evaluated individually for each patient and then averaged across all the participants. The results were compared between our proposed *two-selected channels* configuration and the state-of-the-art *four-temporal channels* setup [15].

### 3.1. Patient-Specific Channel Optimization

As the first step, we implemented the method outlined in Section 2.3 to determine the optimized scalp area for electrode placement, based on the 10–20 system, for each patient, in order to personalize the wearable EEG device.

**Figure 2:**
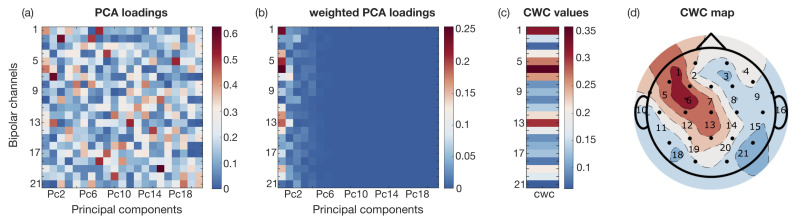
Channel weight coefficient calculation steps. (**a**) PCA loading matrix (L), in absolute value, calculated in a 1-s time segment. *x*-axes represent the PCs, while the *y*-axes show the 21 bipolar channel loadings $l_{ij}$ magnitude according to the colorbar. This map highlights the contribution of each channel in each PC. (**b**) PCA loading matrix of (**a**), where each columns is multiplied by the explained variance associated with each PC. It highlights also the contribution of the PCs to the variance of the analyzed data. (**c**) Weighted mean of the PCA loadings (CWC), obtained by summing up the values in the corresponding row computed in (**b**). The same values are represented in (**d**) using a topographic map. It also serves as a legend for the channel positions of the maps in (**a**–**c**). The dots represent the electrode positions according to the standard 10–20 system. The obtained CWC helps to identify the most significant channels contributing to the recorded neural activity. The example refers to the 1-s time segment of record 3 of patient chb1, extracted during the ictal phase in the δ band.

Figure 2 shows an example of the calculation of the CWCs, highlighting the different steps discussed in Section 2.3.1. Without loss of generality, it specifically refers to the 1-s time segment of record 3 of patient chb01, extracted during the ictal phase in δ band.

In more detail, the map in Figure 2a graphically represents the 21×21 loading matrix (with its elements in absolute value) evaluated by means of the PCA, showing the 21 PCs along the *x*-axis, while the 21 EEG channel labels are displayed along the *y*-axis. To simplify the notation, the bipolar channels of the standard 10–20 system were numbered sequentially from 1 (corresponding to the left frontal region) to 21 (corresponding to the right occipital region) coherently with the topographic map of Figure 2d [27]. Specifically, the numbering follows the following scheme: 1: Fp_1_-F_7_; 2: Fp_1_-F_3_; 3: Fp_2_-F_8_; 4: Fp_2_-F_4_; 5: F_7_-T_7_; 6: F_3_-C_3_; 7: Fz-Cz; 8: F_4_-C_4_; 9: F_8_-T_8_; 10: T_7_-FT_9_; 11: T_7_-P_7_; 12: C_3_-P_3_; 13: Cz-Pz; 14: C_4_-P_4_; 15: T_8_-P_8_; 16: FT_10_-T_8_; 17: FT_9_-FT_10_; 18: P_7_-O_1_; 19: P_3_-O_1_; 20: P_4_-O_2_; and 21: P_8_-O_2_. The loading values associated with each channel for all the evaluated PCs are visualized using a colormap ranging from blue (lowest values) to red (highest values). Following this approach, the 21×21 point map highlights with red points the channels that contribute most significantly to each PC, which stand out from those making a negligible contribution, represented instead by the blue points. Figure 2b shows the updated map obtained by scaling the loading matrix by the explained variance. In this revised map, the colors shift toward blue for higher PCs, indicating that only the first few PCs are most effective in capturing the most relevant information [37,38]. Finally, Figure 2c shows the CWC calculated by summing up the weighted loadings for each EEG channel across all the PCs.

The same values are represented in the map of Figure 2d, which also serves as a legend for Figure 2a–c. In this specific example, it is evident that the analyzed δ activity at this specific instant is localized in region 6, which is covered by the F_3_-C_3_ channel.

**Figure 3:**
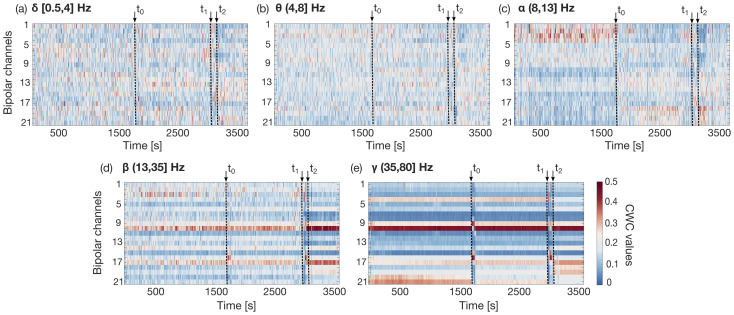
Temporal evolution of channel weight coefficient evolution computed from a typical EEG signal recorded using the standard 10–20 system and decomposed into five clinical frequency ranges (**a**–**e**). The plots refer to record 03 of patient chb01 of the analyzed dataset. *x*-axes represent time in seconds over 1 h, while the *y*-axes show the 21 bipolar channel weight coefficient (defined in the text) values, as indicated by the colorbar in (**e**). For the channels’ positions on the scalp, refer to Figure 2d. These trends illustrate shifts in neural activity across scalp regions as the brain transitions through different phases. Red dotted lines at $t_{1}=2996$ s and $t_{2}=3036$ s denote the start and end of the ictal event, respectively, according to the ground truth. The black dotted line at $t_{0}=1730$ s marks the beginning of the pre-ictal phase, characterized by trend discontinuities.

Repeating the same procedure for the whole record, we obtain the temporal evolution of the CWCs represented in Figure 3 (for the five standard frequency ranges, one in each of the sub-figures, from (a) to (e)). We can notice that higher frequencies more efficiently capture the rapid neuronal discharges that occur in different regions of the scalp during the seizure’s phases. Indeed, as the frequency increases (see Figure 3d,e), two phenomena become evident: some channels tend to dominate others, and trend discontinuities emerge around the 1730th second and the 2996th second, indicated as $t_{0}$ and $t_{1}$ in the plots, respectively. The instant $t_{0}$ exactly corresponds to the onset of the *ictal* phase, as annotated in the dataset, which concludes at the instant $t_{2}$, marking the beginning of a *post-ictal* phase. The discontinuity at instant $t_{1}$, instead, although not annotated in the dataset, could be representative of the onset of a pre-ictal phase. The portion of the record preceding the instant $t_{0}$ will hereinafter be referred to as the *no-seizure* phase, while the segment between $t_{0}$ and $t_{1}$ will be defined as the *pre-ictal* phase [39].

Looking at the temporal trends in more detail, it is possible to observe that while δ and θ waves, shown in Figure 3a,b, respectively, exhibit rather homogeneous activity across channels during all the record, the α band, on the contrary, (Figure 3c), begins to reveal distinct patterns. During the no-seizure phase, in fact, frontal regions 1–4, i.e., channels Fp_1_-F_7_, Fp_1_-F_3_, Fp_2_-F_4_, and Fp_2_-F_8_, are mainly involved in α activity, while in the pre-ictal phase, it shifts to the parieto-occipital regions, mainly focused within 18–20, i.e., around the P_7_-O_1_ channels (see Figure 3c between $t_{0}$ and $t_{1}$). During the ictal phase (between $t_{1}$ and $t_{2}$), α waves are instead predominant in the right temporal regions 16 and 17, covered by FT_9_-FT_10_ and FT_10_-T_8_. Finally, in the post-ictal phase, a configuration similar to the pre-ictal one tends to be reestablished. The β waves (Figure 3d) and the γ waves (Figure 3e) reveal higher channel selectivity during different seizure phases, with γ waves localized more precisely than β.

During the no-seizure phases, i.e., before the instant $t_{0}$, these high-frequency activities are mainly in the left temporal region 10 (T_7_-FT_9_) with notable spots in the right parieto-occipital (21: P_8_-O_2_) and temporal–frontal regions covered by the electrode F_8_, especially in the γ band. Around the $t_{0}=1730$ s, marking the beginning of a pre-ictal phase, the left temporal regions 16 and 9 around the T8 electrode (FT_10_-T_8_ and F_8_-T_8_) become predominant, causing a trend discontinuity in Figure 3d,e. After this discontinuity, the γ waves tend to become dominant in the same regions that characterize the no-seizure phase.

The ictal phase again shows a strong involvement of the right temporal–frontal region (FT_10_-T_8_ and F_8_-T_8_), also extending to the right frontal region (Fp_2_-F_8_ and Fp_2_-F_4_) and right temporal–parietal region (T_8_-P_8_). In the post-ictal phase, after the instant $t_{2}$, high-frequency activity tends to shift again toward the region 10 covered by T_7_-FT_9_. We can observe that γ waves are dominant on the FT_9_ channel throughout the whole considered record, becoming even more pronounced in the post-ictal phase. This effect could be due to individual variations in brain anatomy and functionality, reflecting involvement in cognitive and sensorimotor functions, or latent hyper-excitability, which, while not sufficient to trigger a seizure, could be present at a subclinical level as higher baseline activity [40].

An interesting aspect to observe is that within the individual phases, the trend of CWCs is quite constant. This helps us to effectively condense the information obtained by deriving, for each phase, single values of the CWCs associated with the individual channels. Actually, each temporal phase corresponds to a spatial region that is more or less extended, involving a limited number of channels. With more detail, for each of these phases, a different and localized set of channels tends to emerge as dominant, indicating that it would be theoretically possible to select four representative channels (i.e., one per phase) for a more comprehensive description of the entire seizure cycle. However, since our classification task is formulated as a binary problem (i.e., ictal vs. inter-ictal), we focused on the two phases that are most informative for this purpose: the ictal phase and the no-seizure phase (used to represent the broader inter-ictal class).

This dual-channel strategy enables the model to learn contrasting patterns between seizure and non-seizure activity more effectively than using only one channel while still maintaining a low-complexity configuration. Moreover, the discontinuities present in the CWC time trend enabled us to reduce the inter-ictal phase data to the no-seizure subclass only, allowing for an enhanced distinction between the classes.

**Figure 4:**
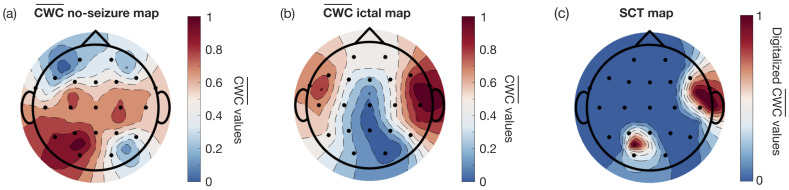
Channel selection method. EEG topographic maps showing the $\bar{\mathrm{CWC}}$ values for each channel, represented in an easily interpretable format. The maps cover the entire scalp, with labeled electrode positions according to the international 10–20 standard system, normalized to the range [0, 1]. They were obtained from all the records of patient chb01 of the dataset. (**a**) $\bar{\mathrm{CWC}}$ values during the no-seizure phase. (**b**) $\bar{\mathrm{CWC}}$ values during the ictal phase. In both maps, areas in red represent higher CWC values according to the colormap. (**c**) XOR map of the digitalized values (with a threshold at 0.9) from (**a**,**b**), referred to as the SCT Map in the text, highlighting the two most significant channels selected in each phase.

The average CWC values obtained by merging the contributions from all the frequency bands and from all the records (following the method described in Section 2.3) are shown in the EEG topographic maps of Figure 4a,b. They specifically refer to no-seizure and ictal phases, respectively. The map in Figure 4a, corresponding to the no-seizure phase, shows that the channel P_3_-O_1_ carries most of the information, while the map in Figure 4b, pertaining to the ictal phase, highlights the channel F_8_-T_8_. This is clearly visible in the *Selected Channels Topological (SCT) Map*, shown in Figure 4c, obtained with the threshold and XOR operations. In the example discussed, the parameter $\bar{\rho }$ calculated between the two identified channels is equal to 0.32, thus indicating a low correlation degree.

Similar temporal patterns are also observed in all the patients within the dataset, although they are not reported here. Therefore, the same procedure was then implemented for all the patients of the dataset, and the results are shown in Table 2. Bipolar channels, corresponding to specific scalp regions, identified during the inter-ictal and ictal phases are specified as Channel A and Channel B, respectively. The $\bar{\rho }$ column reports the correlation values evaluated as described in Section 2.3.2.

It is important to remark that during the implementation of the selection algorithm, careful consideration was given to the fact that the selected channels are used as input to a classifier trained under an LORO cross-validation scheme. Specifically, it was verified that when applying the algorithm to a subset of data obtained by excluding one record from the total number of records for a given patient, the selected pair of channels remained the same across all iterations, regardless of the excluded record. This demonstrates that the selected channel pair does not depend heavily on individual records, ensuring that the classification performance does not drop due to overfitting. The observed consistency in channel selection also indicates that only a few records are sufficient to achieve a reliable and stable selection, thereby simplifying the application of the described workflow. However, for some specific patients, variability in channel selection was observed across the excluded records. In these cases, a majority voting strategy was applied to determine the final channel pair for the subsequent training and testing phases, although the selection was not unanimous.

The exceptions mainly involved patients characterized by a limited number of records (fewer than four) containing ictal events, such as chb04, chb07, chb17, chb19, and chb21, making the selection of channel 2 more challenging. This variability also occurs when the algorithm penalizes channel pairs that exhibit high correlation or that are not exclusively selected from the two distinct classes, as in the case of chb06, consistent with the findings reported in [32]. It is interesting to observe that the presence of multiple active channels for each phase in certain patients may potentially reflect the type of seizure, focal or generalized. However, due to the lack of specific annotations for seizure types within the dataset, a deeper investigation in this regard is not feasible.

We can notice that the $\bar{\rho }$ values, computed between the channels selected for different patients, remain below 0.33 in most cases, indicating minimal overlap in information between the two channels, which is in turn advantageous at the classification stage. The only exception is related to the chb06 and chb21 patients, exhibiting high correlation values (0.47 and 0.43, respectively). This in turn reflects on poor performance at the classification stage, as discussed in the next sections.

### 3.2. Performance Evaluation

Once the channel pairs were selected for each patient, we evaluated the patient-specific classification performance, reporting the obtained results in Table 3. To conduct a comparative analysis with the state-of-the-art and demonstrate the effectiveness of the proposed approach, in the same table, we also included the results obtained using the configuration that takes four fixed channels located in the temporal region as input (F_7_-T_7_, T_7_-P_7_, F_8_-T_8_, and T_8_-P_8_) [15], as described in Section 2.4.1. Both configurations were trained and tested following the same procedure and evaluated using identical metrics, ensuring a fair comparison. This enables us to fairly compare the performance of our two-selected-channels configuration with a four-temporal-channels configuration similar to those adopted in recent works [11,20,35,41]. For both the two-channel and four-channel configurations, the last row of Table 3 reports the average results for each metric for the 24 patients in the CHB dataset. In particular, the last column provides the cumulative results, reflecting the total number of detected ictal events with respect to the total number of annotated events across all the patients (in the form detected/total number). The last two rows summarize the overall achieved results: we can notice that our two-selected-channels-based system successfully detected 152 out of 181 ictal events, with an average delay of approximately 11 s, which is about one-third of the average duration of a seizure calculated within the analyzed dataset. The classification performance yielded an average SP of 1.00 and SN of 0.67, achieving a remarkable bAcc of 0.83. Moreover, the system demonstrated high robustness against false positives, with a low false-positive rate of only 0.10 FP/h. The model obtained for each patient has a size of 51 KB, with approximately 9.5k model parameters, thus making it suitable for its deployment on low-cost microcontroller units.

## 4. Discussion

### 4.1. Overall Analysis

The proposed method enables the calculation of CWCs, offering a quantifiable measure of the relative importance of each channel in datasets acquired through standard EEG systems. This approach has been crucial in identifying the most relevant scalp regions during seizures, thereby facilitating optimized electrode placement for continuous seizure monitoring in a personalized manner. By focusing on channels with the highest CWC values, we effectively isolate those that best capture seizure-related activity while suppressing redundant and noisy signals. Based on linear inter-relationships and orthonormal transformations among the different channels, our method is particularly advantageous for low-dimensional EEG datasets, where conventional analysis techniques often struggle to distinguish between meaningful patterns and irrelevant noise. To the best of our knowledge, no previous study has utilized PCA in this specific, time-aware, frequency-specific, and channel-focused manner. For instance, Li et al. proposed PCA for dimensionality reduction in the features extracted from EEG signals, transforming the original high-dimensional feature space into a smaller set of uncorrelated PCs [29]. Chakrabarti et al. used PCA to select the subset of channels that have the highest correlation with the PC. However, without a thorough analysis across different frequency bands and without a time-aware approach, they were only able to achieve acceptable performance by reducing the number of channels from 23 to 18 [42]. On the other hand, Mateo et al. applied PCA for noise elimination in EEG data [30], demonstrating effectiveness when the noise level is sufficiently low. Intriguingly, the scalp regions identified by our algorithm are largely consistent with those highlighted by a more complex method described in [32], which involves training a CNN architecture that takes 21 channels as input and produces EEG Channel Activation Maps to identify the brain regions activated during seizures. In addition, leveraging its low computational cost, the method processes data on a second-by-second basis, producing dynamic maps that capture the temporal evolution of the observed parameters. These maps offer an effective and intuitive visualization tool for clinical experts, facilitating data interpretation and analysis.

Thanks to this optimization strategy, we developed a system working with only two selected channels, demonstrating greater advantages than the recently analyzed four-channel systems. Selecting more than two channels (e.g., three or four) would introduce increased system complexity and reduced wearability while introducing negligible gain in performance for this specific detection task. On the other hand, using only one channel, although not reported here, results in a noticeable performance drop. The results obtained with the two-channel configuration are broadly comparable to those from the four-fixed-channels setup, with the added benefit of reducing the system’s complexity and footprint. Specifically, we observed no differences in terms of SP, which can be attributed to the inherent imbalance between the ictal and inter-ictal classes in the training and validation datasets. This imbalance naturally results in a negligible number of FPs compared to the high number of TNs. When considering SN, the two-channel configuration achieved a slight improvement of about 1%, reflecting an increased number of detected ictal events (152 vs. 149) while maintaining a comparable average detection delay of approximately 11 s. We explicitly remark that the balanced accuracy value reported in Table 3 is penalized compared to the traditional accuracy calculation, which is instead based on the number of correct predictions divided by the total number of predictions. By calculating the accuracy, we obtain a value of 0.99, strongly influenced by the high number of TNs correctly identified.

More importantly, our two-channel setup demonstrated increased robustness against FPs due to the reduction in noise and redundancy. The average FP rate in the state-of-the-art four-temporal-channels configuration was higher at 0.15 FP/h compared to 0.10 FP/h in the proposed two-channel system, with a non-negligible improvement of 30% for our system, which further confirms the advantage of the proposed optimized approach. Reducing the FP rate is crucial for improving the reliability of seizure detection systems, as minimizing false alarms that can lead to unnecessary interventions, in turn, improves clinical efficiency and patient quality of life by ensuring a more trustworthy and user-friendly solution. Frequent false alarms may also cause alarm fatigue, reducing responsiveness to actual seizures [14,36].

### 4.2. Performance Comparison with the State-of-the-Art

In recent years, several lightweight seizure detection approaches have been proposed, especially with configurations that adopt all the channels provided by standard headsets (for example, following the 10–20 standard). Those works are summarized in Table 4. For instance, LightSeizureNet [32] achieved outstanding performance with an SN of 0.99, while other recent studies such as those reported by Wang et al. [43] and Ke et al. [44] reported SN values above 0.97, confirming the effectiveness of compact architectures when full-channel data are available.

Anyway, it is important to point out that the concept of a lightweight approach extends beyond algorithmic compactness alone. Our focus, in fact, is on the development of a fully lightweight pipeline, which also includes a minimal EEG setup using at most two channels, so as to be easily integrated into a wearable device. In this regard, several strategies have been recently proposed to identify optimized subsets of EEG channels. These include, for instance, techniques based on Locally Adaptive Feature Selection (LAFS) [22], Mutual Information (MI) and RFs [45], Pearson correlation [20], and variance-based ranking [19]. Chakrabarti et al. employed the PCA to select a number of channels varying from a minimum of 4 to a maximum of 22. Their best performance was achieved using an 18-channel configuration, reaching an accuracy of 0.86, compared to our reported accuracy of 0.99 using only two channels. As the number of channels decreased, performance declined, with accuracy dropping to 0.78 when using four channels [42]. In general, methods reporting the highest performance typically rely on a higher number of EEG channels, which compromises their feasibility for wearable applications. For instance, MI- and RF-based approaches reported an SN of 0.86 using five channels [45], a configuration that, although effective, limits applicability in daily use scenarios.

Conversely, when the number of channels is further reduced, a performance drop is often observed. As an example, Gifford et al. [22] used LAFS to select three channels (thus one more than the proposed approach), achieving an SN of 0.65 (vs. 0.67 of the proposed approach), despite using one more channel. Similarly, Shoka et al. [19] adopted a variance-based selection method to identify three channels, yet their reported Acc did not exceed 0.83 (vs. 0.99 of the proposed approach). In this context, our method offers a compelling alternative. By combining patient-specific optimization with an efficient channel selection strategy, we demonstrate that noteworthy performance can still be achieved using two EEG channels alone. For instance, our method allows us to significantly outperform the 0.72 Acc reported by Affes et al. [46] with a system based on two channels. This comparison highlights the effectiveness of our patient-specific two-channel configuration, which not only reduces complexity and enhances suitability for wearable systems but also maintains, and in some cases also improves, detection performance compared to several multichannel solutions.

### 4.3. Patient-Wise Analysis

While the overall results confirm the validity of our approach, a more detailed analysis at the patient level reveals important insights into individual variability and the factors influencing performance.

**Table 4:** Comparison against the existing literature approaches in terms of characteristics and achieved performance. Note that the first set of methods do not apply any selection strategy. In contrast, methods in the second set can be considered lightweight, both in terms of the neural network architecture and the EEG setup, which is suitable for wearable applications. Among these, the only directly comparable method is [46], which uses two selected channels and achieves an accuracy of 0.72, significantly lower than the 0.99 accuracy achieved by our proposed approach.

Reference	Channels	Selection Method	Classification Model	SN	Acc
Qui et al. [32]	23	not implemented	CNN	0.99	N/A
Bahr et al. [47]	23	not implemented	CNN	N/A	0.96
Ke et al. [44]	23	not implemented	VGGNet (CNN)	0.99	0.98
Wang et al. [43]	23	not implemented	PCNN-Bi-LSTM	0.98	0.99
Thuwajit et al. [48]	21	not implemented	EEGNET-8.2 (CNN)	0.81	0.96
			EEGWaveNet (CNN)	0.69	0.98
Chakrabarti et al. [42]	18	PCA	MLP	N/A	0.87
Dokare and Gupta [45]	5-opt.	MI and RF	SVM	0.87	0.98
Amer et al. [20]	4-fixed	PCC	CNN	N/A	0.99
Ingolfsson et al. [15]	4-fixed	not implemented	EpiDeNet (CNN)	0.69	-
Gifford et al. [22]	3-opt.	LAFS	Multi-Head Self-Attention	0.65	0.85
Shoka et al. [19]	3-fixed	highest variance	SVM	N/A	0.83
Affes et al. [46]	2-opt.	Channel Attention-	CGRNN	N/A	0.72
		MLP	(CNN + GRU)		
**Proposed work**	2-opt.	temporal PCA	CNN	0.67	0.99
					0.83 (bAcc)

fixed: fixed channels; opt.: optimized channels; N/A: not available in the original publication. All acronyms are reported in the Abbreviations list at the end of this manuscript.

It is worth highlighting that most studies published in recent years on the same topic have limited their experimental evaluation to a small subset of patients from the same dataset [14,16,25]. In contrast, our study extends the analysis to all available patients, providing a more comprehensive overview of the system’s performance and evaluating its robustness across a diverse range of seizure patterns and patient-specific characteristics.

In this regard, the first section of Table 3 highlights patients for whom the two-channel configuration detects more epileptic seizures than the four-temporal-channels setup, with 40 out of 51 ictal events detected. This improvement corresponds to an average increase in SN from 0.50 to 0.66, which deserves particular attention. The state-of-the-art four-temporal-channel approach relies on fixed electrode positions, which may not capture signals from scalp regions most relevant to the epileptic activity. As a result, the recorded data may be weakly informative, leading to a higher number of FNs and a consequent drop in SN. In contrast, our method selects the two most informative scalp regions for each patient, enabling the classification model to better detect ictal patterns and thus improve SN. For example, in the case of patient chb04, the SN increases remarkably from 0.18 to 0.71 when using the optimized, patient-specific two-channel configuration. For all these patients, the channel selection algorithm identified a single pair of uncorrelated bipolar channels (one emerged from the ictal phase and the other from the no-seizure phase), in alignment with the observation based on activation maps provided in [32]. In two of these six patients (chb13 and chb17), the selected channels do not overlap with those in the four-channel configuration; in the other four cases, only one channel is shared between the configurations. This results subset underscores even more sharply the importance of selecting an optimal channel pair for effective seizure detection, often outperforming systems with a higher number of channels.

The second section focuses on patients where the two-channel configuration performs comparably to the four-temporal setup in terms of event detection. In most of these cases, the four-channel configuration identified all ictal events. However, the reduction in the channel number did not adversely affect the detection capability or sensitivity. For the majority of these patients, the selection algorithm was not critical: it naturally selected a single pair of optimal channels. Conversely, in seven cases (chb02, chb03, chb11, chb19, chb20, chb22, and chb23), multiple channels were identified in both the ictal and no-seizure phases, requiring a correlation analysis to select the optimal channel pair with the lowest correlation coefficient. The identification of multiple potential channel pairs suggests that these patients may experience generalized seizures, although the lack of specific annotations for this type of seizure in the dataset limits further investigation. Nevertheless, even for this subset of patients, an optimized two-channel configuration was identified without compromising performance.

The third section reports the few patients (3 out of 24) for whom the two-channel configuration performed slightly worse than the four-channel setup. For these patients, the model detected fewer seizures and exhibited a lower SN (0.31 vs. 0.54 on average). This reduced performance can be attributed to the complexity of the selection process, which was not straightforward for all patients. In some cases, it was challenging to identify distinct and uncorrelated channels for the ictal and no-seizure phases. For patient chb06, the algorithm identified the two channels as significant in only one phase due to the XOR operation employed, with a high correlation ($\bar{\rho }=0.47$) between the selected channels. This resulted in a reduced number of detected ictal events and lower performance metrics, with the SN dropping from 0.63 to 0.24 and the bAcc decreasing from 0.81 to 0.62. Similarly, patient chb21 also exhibited a high correlation ($\bar{\rho }=0.43$) between the selected channels. These results confirm that selecting channels conveying distinct and non-overlapping information from the ictal and inter-ictal phases is crucial for optimizing classification performance.

Finally, it is important to consider that the complexity of the detected signals, in terms of the number and duration of ictal events, could affect detection performance. For instance, patient chb12 frequently experienced multiple ictal events (two to five) within the same record. This phenomenon was even more evident for patient chb16, where seizure detection was problematic in both configurations due to the very short duration of ictal phases (average 9.00±3.00 s). These two specific cases have been non-trivial also for neurologists as discussed in previous works [25].

Notably, our approach successfully detected all the seizures occurring in the subset of patients considered in [25], including the challenging ones, and outperformed earlier efforts involving expert-driven channel selection and dataset re-annotation.

## 5. Conclusions

We presented an efficient EEG-based seizure detection system that employs lightweight deep learning techniques for real-time, personalized monitoring with minimal invasiveness and computational overhead. By incorporating an efficient patient-specific channel selection method, our approach significantly reduces the number of electrodes required for effective seizure detection while maintaining or even enhancing diagnostic accuracy. Unlike conventional multichannel EEG systems, which are often impractical for continuous monitoring, our system identifies just two personalized channels optimized for each patient’s unique seizure patterns. This advancement aligns with the ongoing trend of minimizing the number of electrodes in non-invasive EEG systems, providing a robust, data-driven framework for optimized electrode placement. A key strength of our system is its interpretability. It generates time-evolving topographical maps that simplify the analysis of seizure-related brain activity. These maps can be easily interpreted by clinicians and directly compared with conventional diagnostic imaging techniques such as Magnetic Resonance Imaging (MRI) or Computed Tomography (CT) scans, laying the groundwork for further research into validating the clinical utility of these maps in seizure monitoring and prediction. The performance of the proposed system was validated through extensive experimentation on the publicly available CHB-MIT EEG dataset, demonstrating its effectiveness. With a balanced accuracy of 0.83 (and an impressive accuracy of 0.99) evaluated at the segment level, our system performs comparably to or even exceeds the performance of existing multichannel systems based on a four-channel configuration designed for wearable devices. Notably, our two-channel system detected 152 out of 181 ictal events, outperforming the four-channel configuration trained and tested with the same method, which detected 149 ictal events. The personalized nature of our system offers significant advantages in terms of comfort, wearability, and long-term usability. By reducing the channels to just two, our system can be seamlessly integrated into lightweight, user-friendly wearable devices. This greatly enhances the feasibility of continuous, real-time seizure monitoring in everyday life. Future work will focus on validating the clinical application of this system and exploring its integration into more efficient seizure prediction and detection frameworks. Since the channel selection process is independent of seizure detection, this approach also paves the way for incorporating more advanced classification models, such as self-attention mechanisms and self-supervised learning, which could further improve detection accuracy and generalization across diverse patient populations.

## Figures and Tables

**Table 1:** Deep learning model architecture.

Layer	Input Shape	Output Shape	Filters	Kernel Size	Parameters
Conv2D	(1,M,1024)	(4,M,1024)	4	1×4	20
BatchNorm2D	(4,M,1024)	(4,M,1024)			8
MaxPool	(4,M,1024)	(4,M,128)		1×8	
Conv2D	(4,M,128)	(16,M,128)	16	1×16	1040
BatchNorm2D	(16,M,128)	(16,M,128)			32
MaxPool	(16,M,128)	(16,M,32)		1×4	
Conv2D	(16,M,32)	(16,M,32)	16	1×8	2064
BatchNorm2D	(16,M,32)	(16,M,32)			32
MaxPool	(16,M,32)	(16,M,8)		1×4	
Conv2D	(16,M,8)	(16,M,8)	16	16×1	4112
BatchNorm2D	(16,M,8)	(16,M,8)			32
MaxPool	(16,M,8)	(16,1,8)		M×1	
Conv2D	(16,1,8)	(16,1,8)	16	8×1	2064
BatchNorm2D	(16,1,8)	(16,1,8)		8×1	32
AverPool	(16,1,8)	(16,1,1)			
Flatten	(16,1,8)	16			
Dense	16	2			34

*M* is the number of EEG channels set to 2 in the proposed *2-selected* configurations with input size 2×1024 and to 4 in the *4-temporal* configuration with input size 4×1024; *T* is the number of time samples.

**Table 2:** Selected channel pairs with corresponding correlation.

Patient	Channel A	Channel B	$\bar{\rho }$	Patient	Channel A	Channel B	$\bar{\rho }$
chb01	P_3_-O_1_	F_8_-T_8_	0.32	chb13	P_4_-O_2_	Fp_1_-F_7_	0.29
chb02	T_7_-P_7_	T_8_-P_8_	0.32	chb14	T_8_-P_8_	Fp_1_-F_7_	0.24
chb03	P_8_-O_2_	T_7_-P_7_	0.29	chb15	Cz-Pz	T_7_-P_7_	0.30
chb04	Cz-Pz	T_8_-P_8_	0.33	chb16	P_7_-O_1_	C_3_-P_3_	0.25
chb05	Cz-Pz	P_8_-O_2_	0.28	chb17	C_4_-P_4_	P_4_-O_2_	0.31
chb06	F_8_-T_8_	T_8_-P_8_	0.47	chb18	C_4_-P_4_	T_8_-P_8_	0.32
chb07	Cz-Pz	P_8_-O_2_	0.32	chb19	Cz-Pz	T_8_-FT_10_	0.26
chb08	P_8_-O_2_	F_4_-C_4_	0.27	chb20	P_8_-O_2_	T_7_-FT_9_	0.21
chb09	P_7_-O_1_	T_8_-P_8_	0.29	chb21	T_8_-P_8_	C_3_-P_3_	0.43
chb10	T_8_-P_8_	T_7_-P_7_	0.29	chb22	F_4_-C_4_	T_7_-P_7_	0.27
chb11	Fz-Cz	T_7_-P_7_	0.29	chb23	P_8_-O_2_	T_7_-P_7_	0.23
chb12	F_3_-C_3_	F_4_-C_4_	0.23	chb24	C_4_-P_4_	F_8_-T_8_	0.31

**Table 3:** Performance comparison between the four-temporal- and two-selected-channels configuration for each patient. The first section lists the patients where the two-selected-channels configuration outperformed the four-temporal-channels configuration. The second section includes the patients with similar performance across both configurations. The final row shows the results (mean ± standard deviation) across all the CHB patients.

Patient	Configuration	Segment Level				Event Level		
		SP	SN	bAcc		Delay [s]	FP/h	Detected Seizures
chb04	4-temporal	1.00 ± 0.00	0.18 ± 0.21	0.59 ± 0.11		40.0 ± 25.5	0.33	2/4
	2-selected	1.00 ± 0.00	0.71 ± 0.27	0.85 ± 0.13		28.8 ± 32.9	0.11	4/4
chb13	4-temporal	1.00 ± 0.01	0.55 ± 0.44	0.77 ± 0.22		9.7 ± 3.0	0.45	9/10
	2-selected	0.99 ± 0.01	0.65 ± 0.31	0.82 ± 0.16		9.7 ± 2.5	0.36	10/10
chb14	4-temporal	1.00 ± 0.00	0.32 ± 0.41	0.66 ± 0.20		6.5 ± 0.58	0.04	4/8
	2-selected	1.00 ± 0.00	0.53 ± 0.31	0.76 ± 0.16		6.6 ± 1.0	0.00	7/8
chb15	4-temporal	1.00 ± 0.02	0.73 ± 0.19	0.87 ± 0.09		11.5 ± 8.2	0.15	19/20
	2-selected	1.00 ± 0.02	0.82 ± 0.16	0.91 ± 0.08		6.7 ± 5.0	0.21	20/20
chb17	4-temporal	1.00 ± 0.00	0.28 ± 0.25	0.64 ± 0.13		39.5 ± 7.8	0.05	2/3
	2-selected	1.00 ± 0.00	0.46 ± 0.10	0.73 ± 0.05		26.3 ± 6.0	0.00	3/3
chb18	4-temporal	1.00 ± 0.02	0.45 ± 0.38	0.72 ± 0.18		13.5 ± 6.6	0.46	4/6
	2-selected	0.99 ± 0.04	0.55 ± 0.31	0.76 ± 0.15		16.2 ± 13.1	0.11	5/6
chb01	4-temporal	1.00 ± 0.00	0.86 ± 0.19	0.93 ± 0.10		9.0 ± 6.9	0.00	7/7
	2-selected	1.00 ± 0.00	0.91 ± 0.10	0.95 ± 0.05		6.4 ± 3.4	0.00	7/7
chb02	4-temporal	1.00 ± 0.00	0.93 ± 0.08	0.96 ± 0.04		11.0 ± 6.6	0.00	3/3
	2-selected	1.00 ± 0.00	0.93 ± 0.09	0.96 ± 0.04		8.3 ± 2.9	0.08	3/3
chb03	4-temporal	1.00 ± 0.01	0.77 ± 0.14	0.89 ± 0.07		17.4 ± 6.5	0.03	7/7
	2-selected	1.00 ± 0.01	0.92 ± 0.11	0.96 ± 0.05		8.0 ± 5.8	0.08	7/7
chb05	4-temporal	1.00 ± 0.00	0.88 ± 0.12	0.94 ± 0.06		9.8 ± 4.0	0.08	5/5
	2-selected	1.00 ± 0.00	0.83 ± 0.14	0.92 ± 0.07		21.8 ± 16.3	0.00	5/5
chb07	4-temporal	1.00 ± 0.00	0.57 ± 0.22	0.79 ± 0.11		14.3 ± 5.7	0.00	3/3
	2-selected	1.00 ± 0.00	0.59 ± 0.31	0.79 ± 0.16		28.3 ± 12.3	0.03	3/3
chb08	4-temporal	1.00 ± 0.00	0.83 ± 0.15	0.91 ± 0.07		11.2 ± 3.27	0.20	5/5
	2-selected	0.99 ± 0.02	0.85 ± 0.07	0.91 ± 0.05		15.2 ± 3.11	0.03	5/5
chb09	4-temporal	1.00 ± 0.00	0.94 ± 0.03	0.97 ± 0.01		7.3 ± 1.7	0.00	4/4
	2-selected	1.00 ± 0.00	0.93 ± 0.03	0.97 ± 0.01		8.3 ± 2.5	0.00	4/4
chb10	4-temporal	1.00 ± 0.00	0.96 ± 0.07	0.98 ± 0.03		6.0 ± 2.3	0.00	7/7
	2-selected	1.00 ± 0.00	0.94 ± 0.08	0.97 ± 0.04		4.9 ± 3.0	0.00	7/7
chb11	4-temporal	1.00 ± 0.00	0.83 ± 0.25	0.91 ± 0.12		3.0 ± 1.7	0.03	3/3
	2-selected	1.00 ± 0.00	0.82 ± 0.08	0.91 ± 0.04		5.0 ± 5.2	0.17	3/3
chb19	4-temporal	1.00 ± 0.00	0.94 ± 0.03	0.97 ± 0.01		9.3 ± 3.8	0.10	3/3
	2-selected	1.00 ± 0.00	0.83 ± 0.02	0.91 ± 0.00		16.7 ± 1.5	0.14	3/3
chb20	4-temporal	1.00 ± 0.00	0.59 ± 0.42	0.80 ± 0.21		11.9 ± 6.9	0.03	7/8
	2-selected	1.00 ± 0.00	0.57 ± 0.21	0.78 ± 0.10		13.9 ± 5.0	0.07	7/8
chb22	4-temporal	1.00 ± 0.00	0.89 ± 0.17	0.94 ± 0.08		12.3 ± 11.0	0.00	3/3
	2-selected	1.00 ± 0.00	0.82 ± 0.12	0.91 ± 0.06		16.3 ± 7.0	0.00	3/3
chb23	4-temporal	1.00 ± 0.01	0.86 ± 0.13	0.93 ± 0.06		12.0 ± 5.9	0.18	7/7
	2-selected	1.00 ± 0.01	0.86 ± 0.05	0.93 ± 0.03		9.1 ± 2.4	0.18	7/7
chb24	4-temporal	1.00 ± 0.00	0.52 ± 0.27	0.76 ± 0.13		9.8 ± 3.2	0.14	13/16
	2-selected	1.00 ± 0.00	0.50 ± 0.30	0.75 ± 0.15		9.08 ± 2.53	0.14	13/16
chb06	4-temporal	1.00 ± 0.00	0.63 ± 0.35	0.81 ± 0.17		7.9 ± 1.7	0.07	8/10
	2-selected	1.00 ± 0.00	0.24 ± 0.32	0.62 ± 0.16		9.0 ± 2.16	0.00	4/10
chb12	4-temporal	1.00 ± 0.01	0.42 ± 0.31	0.71 ± 0.15		11.4 ± 5.9	0.29	20/27
	2-selected	1.00 ± 0.01	0.38 ± 0.20	0.69 ± 0.13		11.6 ± 7.9	0.33	19/27
chb21	4-temporal	1.00 ± 0.00	0.65 ± 0.24	0.82 ± 0.12		14.3 ± 14.4	0.03	4/4
	2-selected	1.00 ± 0.00	0.29 ± 0.28	0.64 ± 0.14		26.0 ± 18.5	0.03	3/4
chb16	4-temporal	0.99 ± 0.05	0.00	0.50 ± 0.00		-	0.88	0/8
	2-selected	1.00 ± 0.00	0.00	0.50 ± 0.00		-	0.00	0/8
**Overall**	**4-temporal**	1 ± 0.01	0.66 ± 0.32	0.83 ± 0.16		11.38 ± 8.02	0.15 ± 0.21	149/181
	**2-selected**	1 ± 0.01	0.67 ± 0.31	0.83 ± 0.16		11.47 ± 9.75	0.10 ± 0.11	152/181

## Data Availability

The analyzed data and the source code are available via a GitHub repository: https://github.com/MiviaLab/INBIT/tree/main/Seizure-detection.

## Abbreviations

The following abbreviations are used in this manuscript:

Acc	Accuracy
AI	Artificial Intelligence
bAcc	Balanced Accuracy
CHB-MIT	Children’s Hospital Boston–Massachusetts Institute of Technology
CNN	Convolutional Neural Network
CT	Computed Tomography
CWC	Channel Weight Coefficient
EEG	Electroencephalography
FN	False Negative
FP	False Positive
FP/h	False Positive per Hour
GRU	Gated Recurrent Neural Network
LAFS	Locally Adaptive Feature Selection
LORO	Leave-One-Record-Out
LSTM	Long Short-Term Memory
MI	Mutual Information
MLP	Multi-Layer Perceptron
MRI	Magnetic Resonance Imaging
PCA	Principal Component Analysis
PC	Principal Component
PCC	Pearson Correlation Coefficient
RF	Random Forest
SCT map	Selected Channels Topological Map
SN	Sensitivity
SP	Specificity
SVM	Support Vector Machine
TN	True Negative
TP	True Positive
